# Reduced Processing of Facial and Postural Cues in Social Anxiety: Insights from Electrophysiology

**DOI:** 10.1371/journal.pone.0075234

**Published:** 2013-09-09

**Authors:** Mandy Rossignol, Sophie-Alexandra Fisch, Pierre Maurage, Frédéric Joassin, Pierre Philippot

**Affiliations:** Institut de Recherche en Psychologie, Université catholique de Louvain, Louvain-La-Neuve, Belgium; Ecole Normale Supérieure, France

## Abstract

Social anxiety is characterized by fear of evaluative interpersonal situations. Many studies have investigated the perception of emotional faces in socially anxious individuals and have reported biases in the processing of threatening faces. However, faces are not the only stimuli carrying an interpersonal evaluative load. The present study investigated the processing of emotional body postures in social anxiety. Participants with high and low social anxiety completed an attention-shifting paradigm using neutral, angry and happy faces and postures as cues. We investigated early visual processes through the P100 component, attentional fixation on the P2, structural encoding mirrored by the N170, and attentional orientation towards stimuli to detect with the P100 locked on target occurrence. Results showed a global reduction of P100 and P200 responses to faces and postures in socially anxious participants as compared to non-anxious participants, with a direct correlation between self-reported social anxiety levels and P100 and P200 amplitudes. Structural encoding of cues and target processing were not modulated by social anxiety, but socially anxious participants were slower to detect the targets. These results suggest a reduced processing of social postural and facial cues in social anxiety.

## Introduction

Social anxiety disorder (SAD) has been characterized by a fear of social situations, often accompanied by their avoidance [1,2]. Cognitive models of social anxiety propose that patients with SAD selectively attend to social threat cues and that this bias would be involved in the development and the maintenance of social anxiety symptoms [3–6]. In this context, many studies have examined emotional biases in SAD by exploring emotional facial expressions (EFE) processing [7–9]. Faces are of particular relevance for individuals with social anxiety since they carry markers of interpersonal evaluation. Authors in this field have accorded a particular interest to threatening faces expressing anger, which represents a direct threat. Accordingly, individuals with social anxiety display an attentional bias, in such a way that they show greater attention to angry faces when presented in competition with neutral ones ( [7,10] ; for a review, see 11). Additionally, SAD has been associated with subsequent difficulties in disengaging attention from threatening faces as compared to neutral ones [12–14]. Moreover, social anxiety individuals may try to avoid threat and look away from threatening stimuli. In line with this suggestion, studies presenting pairs of faces (positive, neutral or negative) and objects reported that socially anxious participants were faster in identifying the probe when it occurred in the location of the objects, suggesting an avoidance of face stimuli [15,16] Avoidance may also reflect a more later stage of cognitive processing of threat. This hypothesis was confirmed in an eye movements study [17] presenting pairs of faces (neutral and positive or negative) to participants reporting low of high fear of social evaluation (FNE). High FNE participants looked at the emotional faces longer during the first second of stimulus exposure, but they avoided these faces in the consecutive time interval from 1 to 1.5 s [17]. These observations support the vigilance-avoidance theory and the idea of enhanced early attentional engagement succeeded by strong attentional avoidance [9,18–20].

Electrophysiology has provided temporal cues of these biases in selective attention processes. First, the P100 component appears enhanced when SAD individuals are confronted with pairs of neutral and angry faces as compared to neutral-happy faces [21]. The P100 component indexes basic visual perceptual processing [22] but enhanced P100 amplitudes have been reported during fearful face perception in the general population [23–26], suggesting enhanced early processing of significant events. Thus the presence of a threatening face in a pair of stimuli seems to capture early visual resources in SAD. The P100 is also sensitive to spatial attention [27–29], as demonstrated through enhanced amplitudes for neutral targets replacing angry or fearful faces as compared to neutral ones [30,31]. In this context, higher P100 amplitudes in response to targets replacing threatening faces in subclinical participants selected for high social anxiety [32] and in patients with SAD [21] as compared to non-anxious participants suggest higher attention towards threat in relation with social anxiety.

Interestingly, when the faces are presented one by one and not in pairs, SAD individuals display enhanced P100 compared to that recorded in non-anxious people in response to natural but also schematic and artificial facial stimuli, irrespective of the emotional expressions [33–37]. This effect has been interpreted as attesting hypervigilance for faces [38], congruently with the findings of increased extrastriate visual cortex activation in social phobics when viewing pictures of faces [39].

The occipital P200 component has also provided evidence of disturbed face processing in social phobia. That component is enhanced during fearful [40] and angry face processing in general population [41], and it has been functionally associated with sustained perceptive processing and mobilization of attentional resources [42–44]. Recent evidences showed larger P200 amplitudes for angry-neutral face pairs selectively in high FNE participants [32], enhanced P200 for angry faces as compared to happy or neutral faces in social phobia [45], and a positive correlation between P200 responses to emotional faces (displaying neutrality, anger, fear, disgust or happiness) and FNE level [36]. Authors have postulated that faces constitute salient stimuli in social anxiety and evoke an enhanced orienting (i.e. higher P1) followed by intensified attentional fixation (i.e. enhanced P2) [36]. Moreover.

In addition to hypervigilance to faces, social phobic individuals may also present attentional biases towards other social cues [4]. Indeed, faces are not the only signal bearing social and emotional messages: Our bodies convey emotional states and social intentions through universally recognized postural attitudes and we are able to decode the attitude conveyed by posture without any facial information [46]. Studies have outlined the similarity of the cognitive processes involved in the processing of facial and postural stimuli, arguing for the inclusion of human bodies in the study of the neurobiology of emotional processing (see 47). For instance, face and body postures evoke a similar P100 component [48] and body postures expressing anger may raise the activity of visual areas [49,50], similarly to results obtained for facial cues [51,52]. Finally, fearful postures and faces seem to elicit a comparable attraction of selective attention resources [46], highlighting the great ability of humans to readily decode emotion-related information conveyed by body postures.

Faces and bodies also evoke a similar N170 response [48]. This temporal-parietal negativity generated in the fusiform gyrus is associated with the structural encoding stage of facial processing [53,54]. The perception of bodies also triggers a N170 component, whose amplitude is smaller than that triggered by faces [55]. However, the enhancement of the N170 response for naked bodies [56] suggests a sensitivity of that component to arousal, congruently with results reporting larger N170 amplitudes in response to angry [57] and fearful faces as compared to neutral facial expressions [25,58,59]. The N170 has a clear interest in exploring face processing in SAD since this component provides information about the nature of face encoding [60]. Several affective disorders affecting facial processing, such as schizophrenia or autism, result in disrupted N170 [61,62]. Two studies have reported larger right temporo-parietal N170 to angry faces in SAD when socially anxious individuals had to explicitly identify the emotional expression [63] and when social anxiety was induced in healthy individuals [64]. However, a number of others studies did not replicate this effect [33,34,37,60,65]. In sum, the influence of SAD on the N170 response and its implications remain largely unknown and require further investigations in order to understand how social anxiety may act on the configural encoding of social stimuli as faces and bodies.

For all these reasons, body postures are stimuli of choice in studying biases towards threatening social stimuli in social anxiety. If SAD individuals are constantly scanning their environment for subtle signs of negative evaluation [3], postures should be of particular relevance and evoke the same enhanced process as facial cues. Moreover, the comparison of faces and postures processing could allow to distinguish what is unique to the process of threatening stimuli (i.e. angry faces and postures) or category-specific stimuli (i.e. threatening faces). However, to our knowledge, there are currently no studies exploring these issues. In that context, the present study compared the performances of participants reporting low or high social anxiety during an attention-shifting task [66] using faces and postures expressing neutrality, happiness or anger. During the presentation of these cues, a target appeared in four possible locations, and subjects had to identify its shape (X or O). We aimed to compare the cognitive stages of faces and bodies processing and their abilities to cue target detection. We also aimed to explore timing of occurrence of processing biases on several ERPs components reflecting perceptive and attentional processes. Hence, we first postulated that faces and postures should evoke a similar P100 but that this component should be increased in socially anxious individuals as they should devote increased visual resources to these stimuli [36,37,60]. Increased attentional capture by cues may lead to higher P200 in SAD participants, and we expected enhanced P100 and P200 amplitudes for threatening as compared to neutral and positive stimuli as well as higher amplitudes of the P100 response for targets appearing with these cues [21]. Second, we investigated whether SAD is associated with greater N170 amplitudes in response to faces and postures, which would indicate a more analytical processing of social cues [60]. Finally, slower reaction times for targets preceded by threatening stimuli could highlight a particular disruption of disengagement process in SAD [66]. 

## Method

### 1: Ethic statements

Participants received details regarding the aims of the study and the procedure to be followed before to give their informed written consent. The study was approved by the Ethics Committee of the Institute of Psychology (Catholic University of Louvain) and was conducted according to the principles described in the Declaration of Helsinki.

### 2: Participants

Thirty-six participants were selected from a group of 250 university students screened using the Liebowitz Social Anxiety Scale (LSAS, [67]). The criteria defined by Liebowitz et al. to define social anxiety levels using the LSAS are the following : 55-65 : Moderate social phobia ; 65-80 : Marked social phobia ; 80-95 : Severe social phobia ; Greater than 95 : Very severe social phobia. Hence, high socially anxious (HSA) individuals (N=18; 10 females) were defined as those scoring 65 or more on the LSAS while the low-anxiety (LSA) individuals (N=18; 9 females) had to score under 45 [68]. Participants were also administered the Trait Anxiety Inventory ([STAI-T, 69]), and the Fear of Negative Evaluation questionnaire (FNE, [70]).

Sample characteristics are reported in Table 1. Four participants had to be excluded because of artifacts during event-related potentials (ERP) recording, so that thirty-two participants remained in the sample. Statistical analyses confirm that HSA scored significantly higher on the LSAS (*t*(30)=10.105, *p*=.000), and that they were more afraid of negative evaluation (*t*(30)=6.524, *p*=.000). However, groups did not differ according to trait-anxiety (*t*(30)=1.366, *p*=.183). All volunteers were native French speaking, right-handed, between the ages of 18 and 23 years (no age difference between groups, *t*(30)=.673, *p*=.506), with normal/corrected vision, and no history of psychiatric or neurological disorders.

**Table 1:** Participants’ characteristics as a function of group assignment (standard deviations in parentheses).

	LSA (N=16)	HSA (N=16)
Age	20.4 (3.4)	21.0 (2.7)
Ratio male/female	8/8	8/8
LSAS	32.1 (13.6)	79.3 (12.2)
STAI-B	48.7 (2.5)	50.4 (3.8)
FNE	5.1 (6.1)	15.4 (1.4)

### 3: Procedure

**Figure 1:**
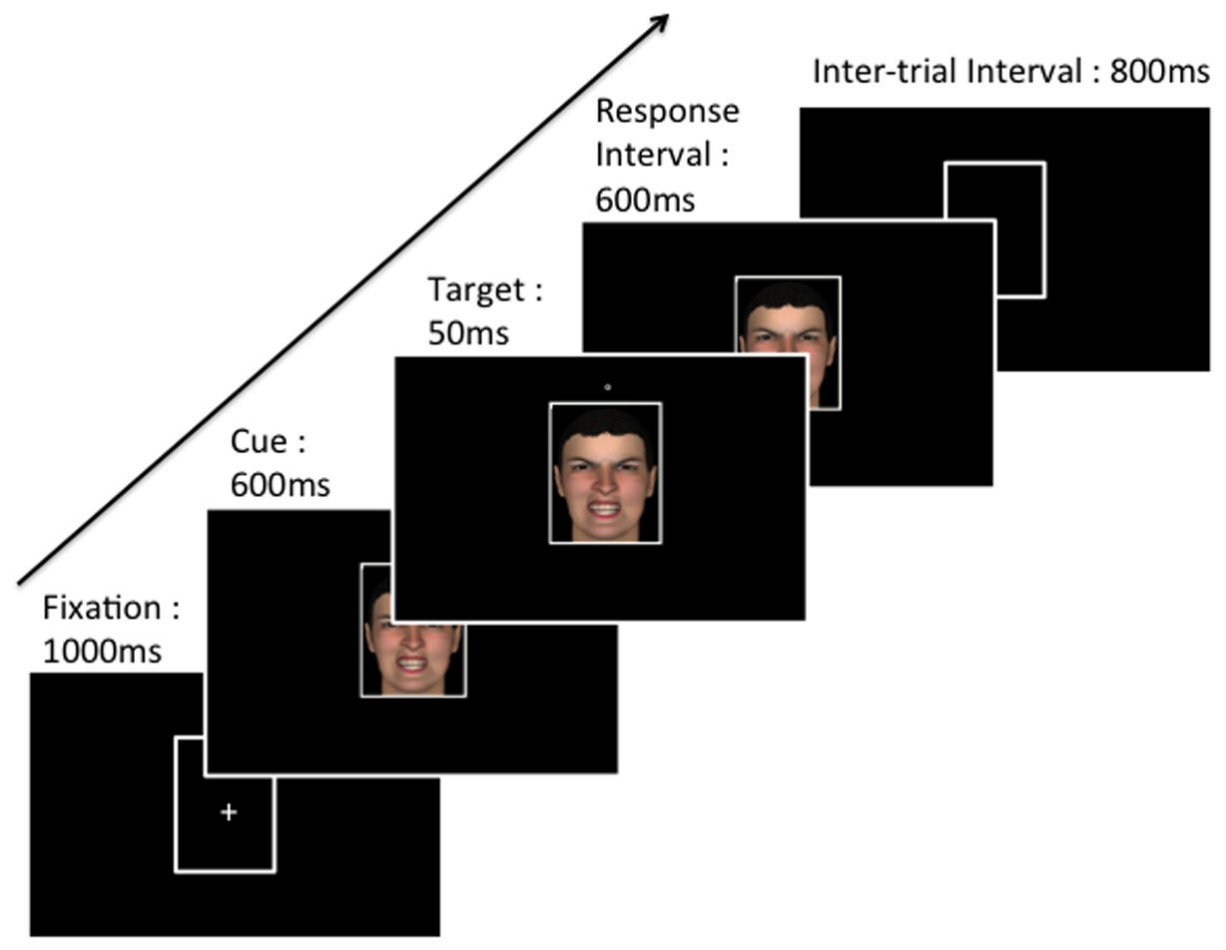
Sequence of events during the task.

**Figure 2:**
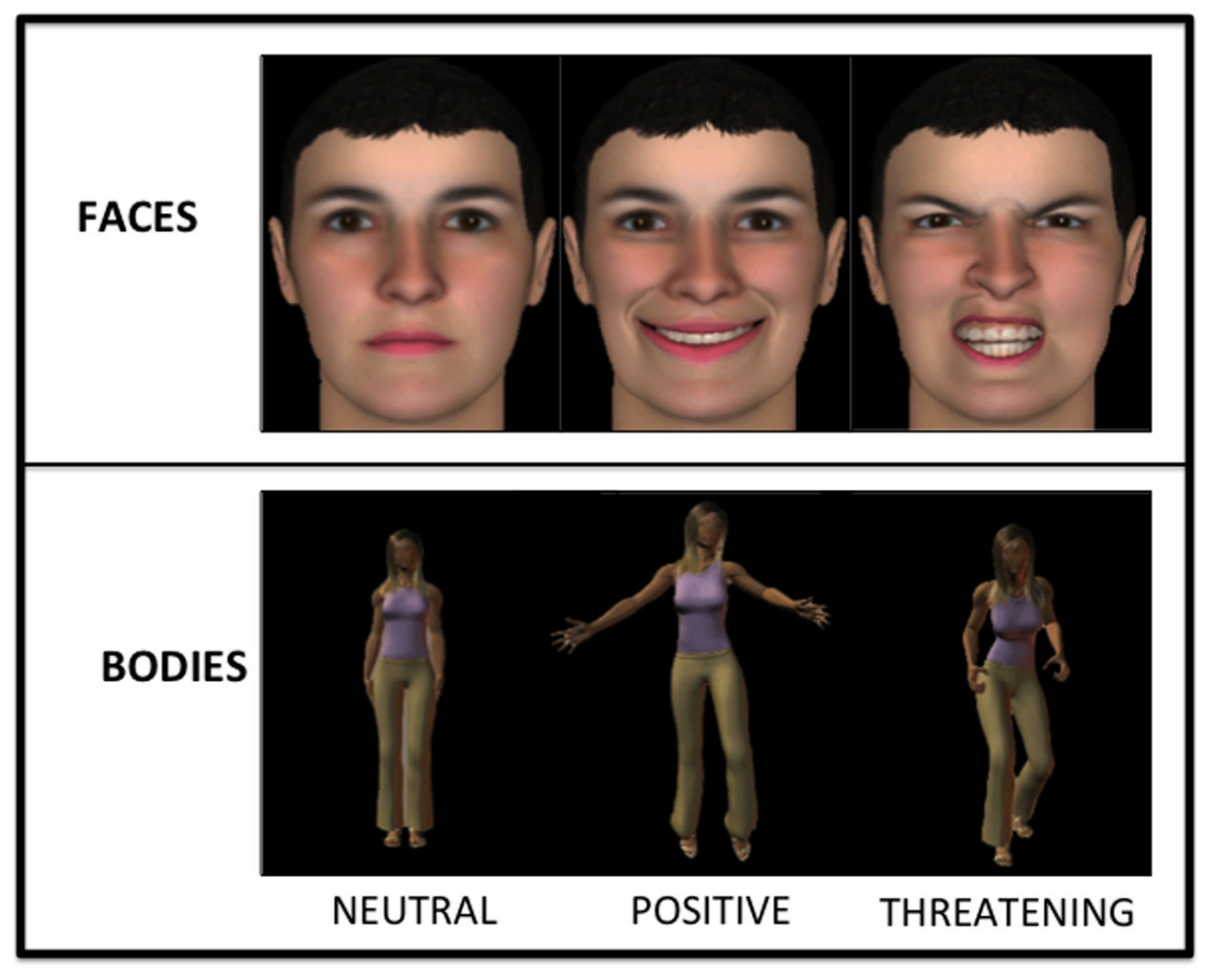
Example of neutral, positive and threatening faces and body postures used as cues.

The experimental procedure used an ‘attention-shifting paradigm’, similar to the one used by Bar-Haim et al. [66] (see Figure 1). Each trial started with the presentation of a fixation display on a black background, composed of a white cross in the centre of an outline frame drawn with white 2-pixel strokes 4x6cm, for 1000ms. A cue-stimulus then appeared inside the frame for a duration of 1250ms. The stimulus set was composed of pictures of human faces and postures (see Figure 2). Facial and postural stimuli were artificial pictures taken from Maurage et al. [71]. They represented two males and two female individuals, expressing neutrality, anger, or happiness (more information about the creation and the validation of these stimuli can be found in [71]). The entire experiment contained 24 cue-stimuli (12 faces: 2 gender x 2 identity x 3 emotions; 12 postures: 2 gender x 2 identity x 3 emotions). After 600ms, a target occurred for 50ms in one of the four possible locations, and was centred above, below, on the left or on the right of the centre of the computer screen. Target stimuli were a white shape, either a cross or a circle, and target shapes and locations were equally probable and their presentation randomized between trials. The cue remained on the screen for an additional 600ms, during which time the participants responded. Finally, after the cue disappearance, the empty frame remained on the screen for 800ms, constituting the inter-trial interval.

A practice bloc of 20 trials alternating the different types of cue stimuli was performed before the beginning of the experiment. Participants were then presented with the four experimental blocs. Each bloc comprised one type of cue-stimuli defined by the following variables: type (faces, postures) and gender (i.e. Bloc 1: female facial cues ; Bloc 2: male faces ; Bloc 3: female bodies ; Bloc 4: male bodies). Each bloc comprised 96 trials and was offered twice in a randomized order, so the entire experiment comprised 768 trials and lasted 30 minutes.

Participants were instructed to focus their gaze on the stimuli presented in the centre of the screen, and then to identify the shape of the target, which could appear in four different locations. Half of the participants had to press “1” in the response box with their left forefinger if the target was a “X”, and “2” with their right forefinger if it was a “O”, and half of participants received the reverse instruction. Participants were informed they could perform the task without ocular movement, and they were asked to refrain from making eye movement. They sat in a chair in a dark room with their head placed 1 m from the screen and restrained in a chin rest. The entire experiment took approximately 50 minutes per participant.

### 4: ERP Recording and data analysis

The electroencephalogram (EEG) recordings were performed with 32 electrodes mounted in an electrode Quick-Cap with the standard 10-20 International System and intermediate positions. Recordings were made with a linked mastoid physical reference, but were re-referenced by using a “common average” [72]. The EEG was amplified by battery-operated A.N.T. ® amplifiers with a gain of 30,000 and a band-pass of 0.01–100 Hz. The impedance of all electrodes was kept below 5kΩ. The EEG was continuously recorded (sampling rate 500 Hz, Eeprobe software, A.N.T.) and the vertical electrooculogram (VEOG) was recorded in a bipolar manner from electrodes placed on the supraorbital and infraorbital ridges of the left eye. Approximately 15% of trials were contaminated by EOG artifacts, which were manually eliminated offline using the procedure developed by Semlitsch et al., which consists of subtracting an average artifact response for each participant based on a percentage of the maximum eye movement potential recorded on Fp1, Fpz and Fp2 prefrontal electrodes [73]. A baseline correction was computed using a 100 ms interval. Epochs beginning 100 ms prior to stimulus onset and continuing for 800 ms were created. Codes synchronized with stimulus delivery were used to average selectively the epochs associated with different stimulus types. Data were filtered with a 30 Hz low-pass filter.

**Figure 3:**
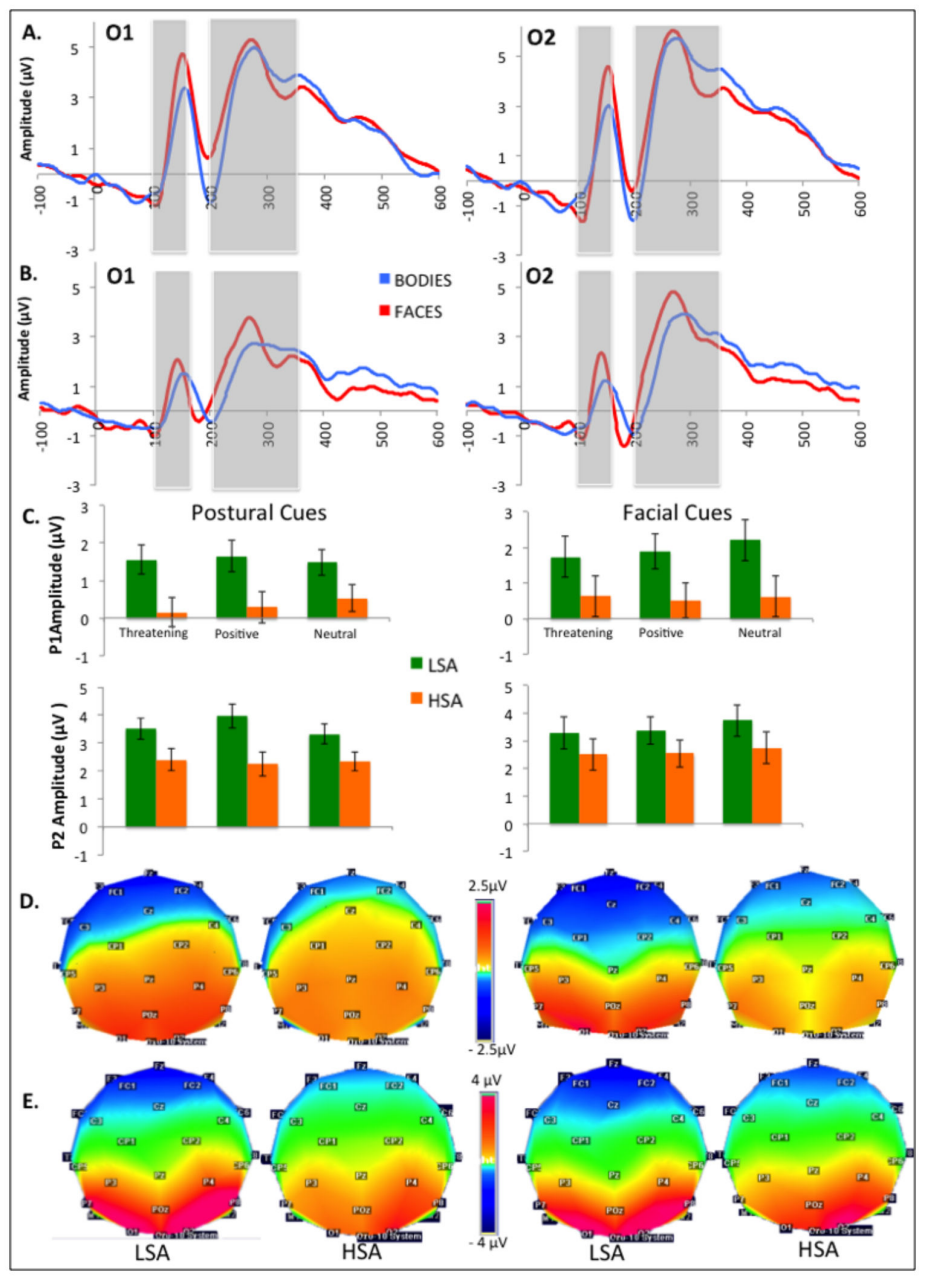
Effects of experimental factors on P100 and P200. A. Grand mean baseline-corrected ERP time courses at O1 and O2 averaged for LSA (A) and HSA (B) in response to facial and postural cues (time windows for P100 and P200 averages are indicated by grey boxes). C. Mean P100 and P200 amplitudes and error bars for the different categories of postural and facial cues. D. and E. Scalp topographies of P100 (D) and P200 (E) for postural (left panel) and facial cues (right panel).

**Figure 4:**
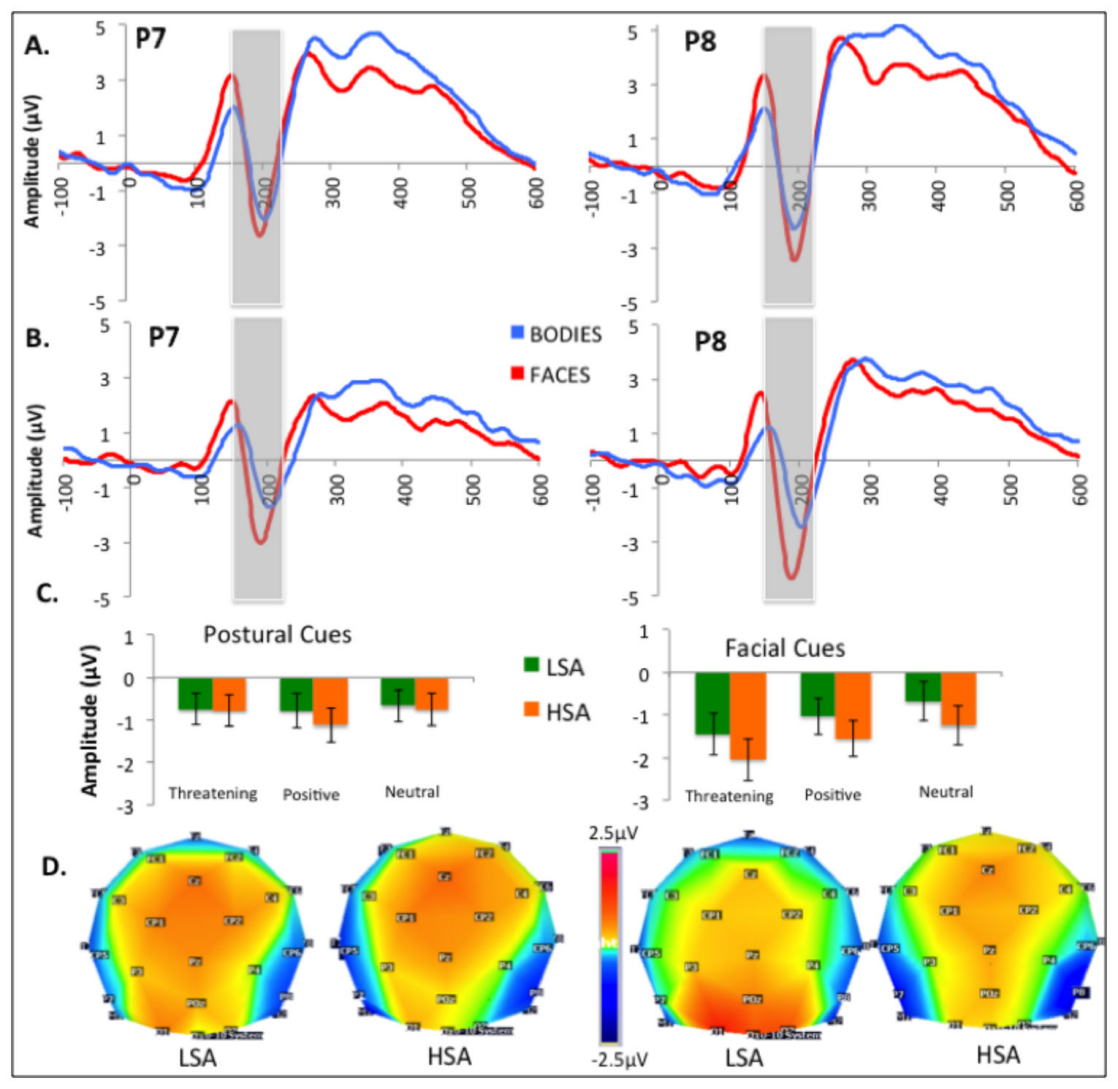
Effects of experimental factors on the N170. A–B. Grand mean baseline-corrected ERP time courses at P7 and P8 averaged for LSA (A) and HSA (B) in response to facial and postural cues. B. Mean N170 amplitudes and error bars for the different categories of postural and facial cues. C. Scalp topographies of N170 for postural (left panel) and facial cues (right panel).

**Figure 5:**
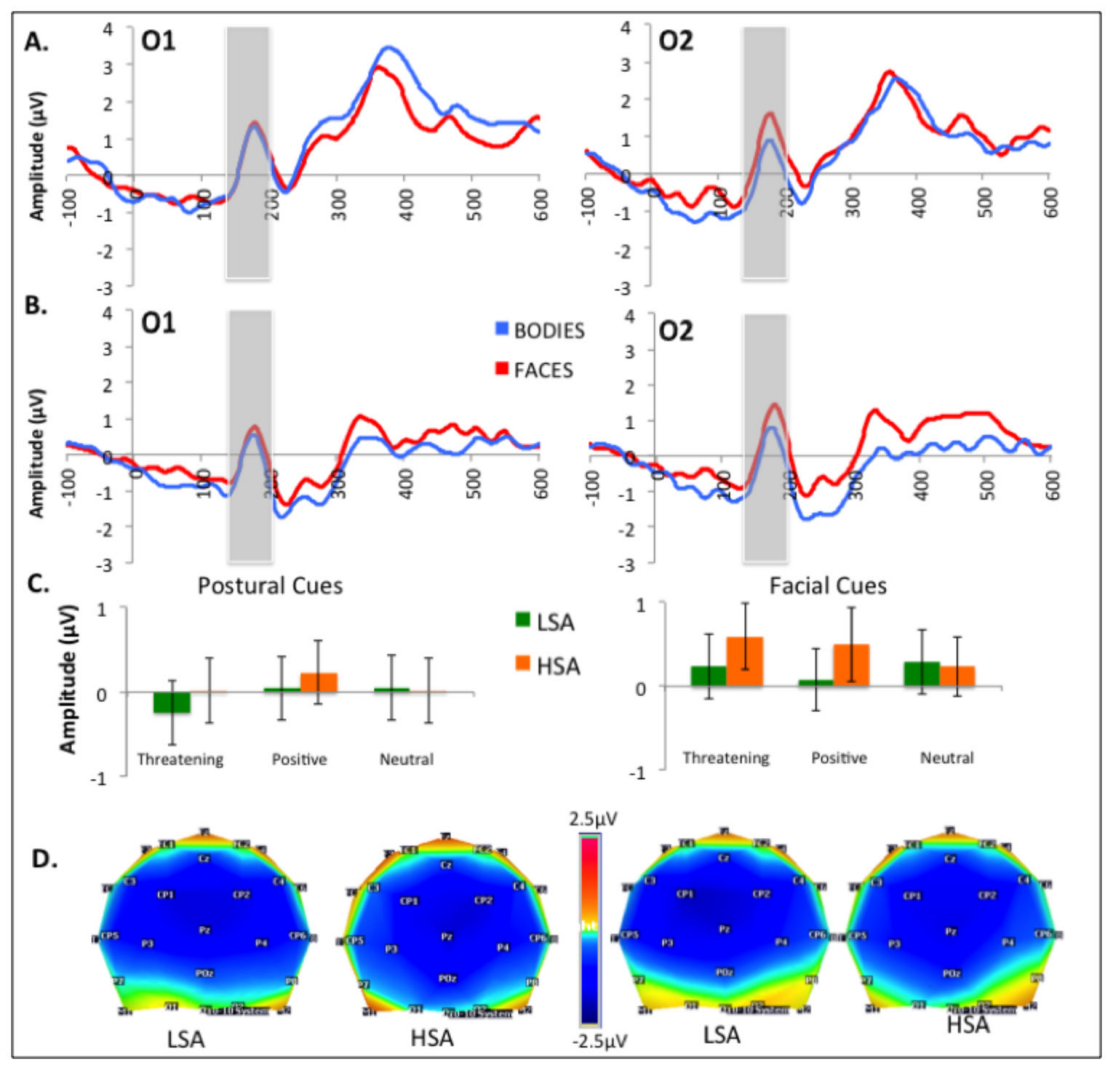
Effects of experimental factors on the P1 in responses to targets. A–B. Grand mean baseline-corrected ERP time courses at O1 and O2 averaged for LSA (A) and HSA (B) in response to the targets cued by postural and facial stimuli. B. Mean P100 amplitudes and error bars for the different categories of targets. C. Scalp topographies of P100-target for postural (left panel) and facial cues (right panel).

Analyses focused on the ERP components elicited by cues and targets, separately (see 66,74). The overall averaged ERPs were examined to define temporal windows on interest electrodes kept constant for all conditions and participants, and mean amplitudes were calculated for each defined window. For cue-evoked components, three ERPs described in the literature focusing on cued-target designs were selected for the analyses: (a) P100, the first positive deflection occurring on occipital sites between 100 and 160 ms after the cue presentation and measured on O1 and O2; (b) the N170, a positive negative deflexion peaking at lateral parietal sites between 150 and 220ms and measured on P7 and P8 ; and (c) the P200, the second positive deflection appearing between 200 and 350ms after face presentation on O1 and O2. For the target processing, analyses were performed on O1 and O2 electrodes within a 140-200ms post-stimulus window to observe the P100 component (the topographic map voltages are shown in Figures 3, 4 and 5). Since the P300 component is involved in the process of response preparation, ERP mean amplitudes were computed in the time window corresponding to P300 (200-400ms after target occurrence). However, social anxiety did not influence P300 amplitude (*F*(1,30)=.000 *p*=.991, *η* ^2^=.000), congruently with recent results [30,32,33,56], so we decided not to detail these analyses in a concern of brevety.

Statistical analyses were computed using Statistical Package for Social Sciences, 17th version (SPSS 17.0). Behavioural data (see table 2) were analysed through 2 (Cue) x 3 (Emotion) x 2 (Group) ANOVA designs. Greenhouse-Geiser epsilon correction was used to compensate for violation of sphericity when appropriate. In the first step, the behavioural data (accuracy and response latencies) and the mean amplitudes of the ERPs were subjected to repeated measures analysis of variance (ANOVA) with Group (LSA and HSA) as the between-subjects factor, and Cues (faces or bodies), Emotion (neutral, positive or threatening) and Hemisphere of recording (right-left, only for the analyses on electrophysiological data) as within-subjects factors. The reported p-levels were corrected for violations of the sphericity assumption using the Greenhouse-Geisser epsilon correction. Simple effects were explored throughout, and a Bonferroni correction for multiple comparisons was applied to all the t-tests.

**Table 2:** Mean correct response latencies and response accuracy and as a function of Group (Standard deviation between brackets).

		Response Accuracy (%)		Response latencies (ms)	
		LSA	HSA	LSA	HSA
Postures	Neutral	80.1 (7.6)	76.3 (8.9)	502 (44.7)	537 (42.3)
	Happy	78.5 (9.3)	72.9 (8.5)	503 (46.7)	544 (40.9)
	Angry	79.7 (9.1)	76.9 (7.7)	501 (44.8)	539 (45.5)
Faces					
	Neutral	84 (7.0)	80.5 (7.7)	504 (48.5)	535 (40.4)
	Happy	77.9 (10.7)	76.7 (6.9)	504 (43.2)	540 (43.5)
	Angry	81.1 (9.2)	78.3 (8.1)	503 (50.3)	533 (38.7)

In the second step, Pearson correlation coefficients were used to explore the relation between the psychometric factors (LSAS, FNE and STAIT scores) and the ERP parameters (P100, N170, P200 and P100-target amplitudes).

## Results

### 1: Behavioural Performance

Correct responses: Analyses did not reveal main effect of Cue (*F*(1,30)=2.986, *p*=.094, η^2^=.091). However, Emotion significantly influenced performance (*F*(2,60)=16.488, *p*=.000, η^2^=.355): targets occurring with positive stimuli were less efficiently detected (76.5%) than ones accompanied by neutral (80.2% - *p*=.000) or threatening stimuli (79.0% - *p*=.000). The comparison between neutral and threatening stimuli did not reach significance (*p*=.081).

However, this effect was expressed differently amongst facial and postural cues, as indicated by a significant interaction between Cue and Emotion (*F*(2,60)=4.078, *p*=.023, η^2^=.120). To follow up this interaction, separate ANOVAs were conducted for facial and postural Cues. Concerning facial cues, the Emotional effect (F(2,62)=15.684, *p*=.000, η^2^=.342) revealed significantly better performance for neutral cues (82.3%) as compared to threatening (79.7%, *p*=.002) and positive faces (77.3%, *p*=.000), and higher performance for threatening as compared to positive faces (*p*=.013). Concerning body postures, the emotional effect (*F*(2,62)=6.588, *p*=.003, η^2^=.183) also revealed lower performance for positive postures (75.7%) as compared to neutral (78.2%, *p*=.008) and threatening ones (78.3%, *p*=.001), but without difference between these last emotional categories (*p*=.832).

Group did not influence correct response production (*F*(1,30)=1.750, *p*=.196, η^2^=.055) and did not interact with Cue (*F*(1,30)=.320, *p*=.576, η^2^=.011) or Emotion (F(2,60)=.260, *p*=.748, η^2^=.009).

Response latencies (on correct responses): The 3 x 2 x 2 ANOVA alone displayed a main effect of Group (*F*(1,30)=5.306, *p*=.028, η^2^=.150), highlighting slower response in HSA as compared to LSA (538.2 vs 503.1ms). No other significant main effect or interaction was found.

### 2: P100 locked on cue occurrence

Analyses displayed a main effect of Group (*F*(1,30)=4.596, *p*=.040, η^2^=.133) revealing reduced P100 amplitudes in HSA (.457µV) as compared to LSA (1.756µV) (see Figure 3).

No other significant main effect or interaction was found.

### 3: N170 locked on cue occurrence

Cue modulate N170 amplitude (*F*(1,30)=6.762, *p*=.014, η^2^=.184), with enhanced waves recorded in response to faces (-1.324µV) as compared to body postures (-.804µV) (see Figure 4).

The N170 was also modulated by Emotion (*F*(2,60)=7.876, *p*=.001, η^2^=.208): Neutral cues evoked reduced amplitudes (-.831µV) as compared to positive (-1.114µV, *p*=.046) or threatening cues (-1.247µV, *p*=.002 - no differences between positive and threatening cues, *p*=.586).

An interaction between Cue and Emotion (*F*(2,60)=8.781, *p*=.001, η^2^=.226) revealed that the emotion effect was not present for body postures (*F*(2,62)=1.983, *p*=.146, η^2^=.062) but well for facial cues (*F*(2,62)=12.976, *p*=.000, η^2^=.295): Angry faces (-1.732µV) elicited a more negative N170 than happy (-1.281µV, *p*=.019) and neutral faces (-.958µV, *p*<.001 - differences between happy and neutral faces, *p*=.062).

Other main or interactional effects did not reach significance.

### 4: P200 locked on cue occurrence

Analyses first displayed a main effect of Group (*F*(1,30)=4.289, *p*=.047, η^2^=.125) revealing reduced P200 amplitudes in HSA (2.466µV) as compared to LSA (3.533µV) (see Figure 3).

Second, a main effect of Hemisphere (*F*(1,30)=10.808, *p*=.003, η^2^=.265) showed higher amplitudes on the right hemisphere (3.287 vs. 2.712µV).

Finally, a interaction between Cue and Emotion (*F*(2,60)=3.754, *p*=.029, η^2^=.111) was decomposed further to show no emotion effect on postures (*F*(2,60)=1.731, *p*=.186, η^2^=.050) but a significant influence of Emotion on faces (*F*(2,60)=3.704, *p*=.030, η^2^=.110) with higher responses for neutral (3.240 µV) as compared to angry faces (2.898 µV, *p*=.023).

### 5: P100 locked on target onset

First, a main effect of Cue (*F*(1,30)=4.806, *p*=.036, η^2^=.138) indicated that enhanced amplitudes were produced for targets accompanied by a facial cue, as compared to a body posture (see Figure 5).

Second, a main effect of Hemisphere (*F*(1,30)=6.777, *p*=.014, η^2^=.184) showed higher amplitudes on the right hemisphere.

However, these effects were further qualified by a significant interaction between Cue and Hemisphere (*F*(1,30)=8.166, *p*=.008, η^2^=.214), that was decomposed into separate ANOVAs for faces and postures. P100 amplitudes were higher on the right hemisphere for faces (*F*(1,31)=10.988, *p*=.002, η^2^=.262) but not for bodies (*F*(1,31)=2.708, *p*=.110, η^2^=.080).

Other main and interaction effects did not reach significance.

### 6: Correlations

Analyses revealed positive correlations between the mean amplitudes of the ERP components (see Table 3). A negative relation between P100 and P200 amplitudes and LSAS scores confirms the above-mentioned result of decreased early perceptual responses to cues when the social anxiety scores augmented. Moreover, accuracy was correlated to the mean amplitude of the P100 for targets, while the response latencies were positively linked to the FNE scores.

**Table 3:** Pearson’s correlations and level of significance (*.05; ** .01; ***.001) between behavioural data, mean amplitudes of ERP waves and psychological variables.

	P100	N170	P200	P100t	RT	CR	LSAS	STAIB	FNE
P100		.436*	.416**	.423*	.078	.324	-.398*	-.145	-.184
N170	.436*		.035	.274	.228	.128	-.172	-.196	-.086
P200	.416**	.035		.171	-.075	.336	-.377*	.109	-.291
P100t	.423*	.274	.171		.099	.401*	-.028	-.275	.063
RT	.078	.228	-.075	.099		-.235	.263	-.85	.464**
CR	.324	.128	.336	.401*	-.235		.019	.279	-.035
LSAS	-.398*	-.172	-.377*	-.028	.263	.019		.388*	.706***
STAIB	-.145	-.196	.109	-.275	-.085	.279	.388*		.072
FNE	-.184	.086	-.291	.063	.464**	-.035	.706***	.072	

## Discussion

The present study intended to compare faces and bodies processing in individuals reporting low or high level of social anxiety to question the specificity/generality of cognitive biases towards social stimuli. We also aimed to explore perceptive and attentional mechanisms involved in the processing of these cues, as well as their abilities to trigger targets detection.

Our first main result relates to the reduced processing of bodies and faces in social anxious individuals as compared to non-anxious participants: P100 amplitudes were negatively correlated to LSAS scores, the more anxious participants displaying smaller P100 responses in response to facial and postural cues, regardless of their emotion load. This result is inconsistent with our hypothesis and the recent literature showing increased amplitude of the P100 component in SAD [21,36,37,60,63,75]. However, the face stimulus was the object to process in these studies, either because a response was required to the stimulus itself (explicit emotional judgments [33] or implicit processing of emotional load in Stroop or Flanker tasks [63,76]) or because the face cued the location of a subsequent target [21,36]. In all cases, subjects had to allocate their attention to facial stimuli to achieve the task. In the present study, the stimuli presented in the fixed central rectangle did not cue the position of the target and, although the instructions asked participants to keep their gaze fixed at the centre of the screen, social anxious participants may have allocated fewer cognitive resources to the process of non-informative cues.

Interestingly, our results outline the same diminution of P100 amplitudes in response to facial and postural cues in SAD, followed by a marked reduction of the P200 component without influence of category or emotional load. The occipital P200 has been functionally associated with sustained perceptual processing [42,43] and the mobilization of attentional resources on salient stimuli to process [77]. Recent results have highlighted social anxiety influence on that component whose enhancement, also evidenced in high trait anxiety [66,77], would reflect a greater mobilization of cognitive resources on motivationally significant stimuli [32,36,45]. The reduction of P200 amplitudes conversely indicates that HSA participants allocated a reduced amount of attentional resources to cues processing, as if they retain to process these task-irrelevant cues. Two hypotheses may be developed to explain that result: first, one may argue that HSA participants allocated less attentional resources to task-irrelevant cues, regardless their social nature, to centre on the primary task. Second, participants may have shifted away from these cues because of their social load. The absence of non-social stimuli in our study prevents us to decide between these hypotheses, and future studies should consider exploring the hypothesis of specific modulations of social stimuli processing vs. a more general pattern involving all kind of visual information.

Beside these reductions of P100 and P200 amplitudes, SAD did not influence the N170 component. Indeed, most of the ERP studies did not observe N170 modulation in SAD [33,35,37,60] and one may hypothesize that the modulation of the N170 by SAD depends on an explicit recognition of threatening faces, since studies showing that effect used explicit recognition tasks [63,64]. However, a recent study has invalidated this hypothesis by showing no modulation of the N170 when SAD individuals performed an adapted emotion Stroop paradigm requiring an implicit or an explicit processing of neutral, positive and threatening faces [60]. Rather, the activation of the neural network underlying face processing, including the fusiform and inferior occipital gyrus, may depend on the task demands and notably, on the requirement of a configural or a featural processing strategy [78]. Finally, the N170 does not reflect the same level of visual process as those objectified by eye-tracking procedure, since studies using eye-tracking technique consistently report an abnormal visual processing of faces in social phobia [14,75,79,80]. Indeed, the N170 indexes a very early stage of face processing, where the face is recognized as such through the encoding of its visual features. That structural processing can be disrupted in some psychopathological states associated with perceptual deficits such as autism or schizophrenia [61,62], which are also associated with disturbed visual scanning [81]. However, the visual scanning of faces or other objects may be independent of their structural encoding, which occurs at a very early and automatic stage. According to that view, it is possible that the structural analysis of faces takes place normally in SAD, to be followed by hyper-scanning of faces after some hundreds milliseconds of presentation and finally an avoidance in the time interval from 1 to 1.5 s after face presentation [75].

Unlike the results of studies using adapted dot-probe or spatial-cueing paradigms [21,32], SAD did not act on the P100 produced in response to target occurrence. The nature of our paradigm may explain these discrepancies. Indeed, in a spatial-cueing paradigm, participants have to use the cue stimuli to facilitate target detection. Consequently, they have to engage their attention towards the cue, and then either keep their attention on the attended location for the valid trials, or disengage their attention to refocus it at the non-cued location to process invalid trials. In the present case, the cue remains present during the whole trial while the target only appears for 50ms. This task requires participants to process a new item of information while already dealing with a first one and consequently to share visual processing resources [66]. It is therefore a measure of cognitive flexibility integrating several attentional components and notably attentional shift and re-engagement [13]. We could have postulated that anxious participants allocated reduced resources to cues processing to better focus on targets. In that case, attention on the primary task would have been associated to enhanced P100 responses to targets and possibly with higher rates of correct responses. However, results show that reduced resources to cues processing were not overcame by increased orienting towards cues. In other words, anxious and non-anxious participants did not differ in the re-engagement of attentional resources towards the targets, indexed by comparable P100 amplitudes regardless of the nature or the emotional load of the cues.

Moreover, high anxious participants were not more efficient to achieve the task: the percentage of correct responses was equivalent for LSA and HSA. Conversely, while response latencies were only calculated on correct responses, social anxious participants detected the targets significantly more slowly, without influence from the nature of the cues or their emotional load. These results are congruent with those of Bar-Haim et al. [66] who reported slower RTs in high anxious individuals as compared to low anxious individuals regardless of facial expressions. These authors interpreted that effect as reflecting increased attentional dwelling on the face cues in high trait anxiety, also indexed by enhanced P200 amplitudes. However, that hypothesis does not apply here since longer response latencies in SAD participants were preceded by reduced processes of cues. Interestingly, correlation analyses show that the mean RTs are directly related to the anxiety of evaluation score. Conversely, the response latencies were not correlated to the amplitudes of the P100 response to cues or targets, nor to the LSAS scores. One may therefore postulate that the initial, automatic processing of cues is under the automatic influence of social anxiety, while the production of a hehavioural response is more influenced by the degree of fear of negative evaluation. It seems that the more subjects are afraid of a negative evaluation, the longer is their response time, a finding consistent with the literature [82,83]. One may also postulate that anxious feelings during the task disrupted performance due to a psychomotor retardation (e.g. freezing) [2]. In that case, cognitive motor processes could by delayed and give rise to a slower answer. Further investigations are needed to examine the origin of that delay by exploring mental chronometry of late conscious and strategic processes. Eye-tracking studies could also offer information about the visual scanning of cues and the subsequent management of targets. Indeed, one study suggested that individuals reporting a fear of social evaluation looked longer at an emotional face during the first second of presentation, but avoided looking at the face in the next interval [75]. A reduced processing of facial and postural cues may have hindered the detection of the targets, which are presented very briefly. Another hypothesis supposes a desire to ensure the exactness of their answers before providing them, as supported by the self-verification model of social anxiety [84].

Interestingly, we did not observe a modulation of social anxiety effects by the cues. P100 and P200 were similarly reduced for faces and bodies in SAD participants, and they were slower than non-anxious participants to answer to both categories of cues. It may mean that social anxiety equally disturbs the processing of emotional information conveyed by faces and body postures [85], but one may not exclude the hypothesis of a more generally disturbed process. Conversely, faces and bodies evoke different perceptive and attentional responses: the N170 was enhanced for faces and emotional influence was only present for facial stimuli on the N170 and the P200. Faces also triggered enhanced P100 in response to targets but without any influence on response latencies. These results suggest that faces and human bodies are not processed similarly in the brain, and that emotional states are more considered on faces, perhaps because of their high representation or cultural habits. However, faces and bodies have comparable abilities to trigger attention since the final performances were comparable for targets cued by facial or postural stimuli.

If the present study is the first to question the cognitive processing of bodies in SAD, some issues should be clarified in future works. First, our design confronted participants to a performance situation, which may have led to focus on the primary task at the cost of cues processing. As we aimed to compare faces and bodies processing, we did not include non-social cues and we cannot disentangle the role of task contingencies and of cues nature on the observed reduced processing. Hence, further studies should deepen these results by including control conditions with non-social objects and specify this diminished process by comparing informative and non-informative cues, in paradigms with and without temporal gap between cues and targets. Second, we asked the participants to refrain making eye movements to detect the targets, but we did not control they respected that instruction using an objective measure as eye-tracking, which could have provided interesting cues about stimuli visual scanning. Finally, participants were selected for their high vs. low scores on the LSAS. That inventory evaluates two core components of social anxiety, namely anxiety and avoidance. Selected HSA participants met the DSM criteria for social phobia, but we excluded individuals with very high scores of trait-anxiety (up to 65) to avoid the problem of comorbidity. However, positive correlation between trait and social anxiety is usual in patients with social phobia [86] and one may question the generalization of our results to that overall population.

In summary, the present study aimed to explore the influence of social anxiety on the processing of social postural and facial cues. Our results show that social anxiety is associated with reduced processing of task-irrelevant postures and faces (diminished P100 and P200 amplitudes). The lack of modulatory effect of cue category or emotional load argues in favour of the automatism of this mechanism, which occurs before the identification of the stimulus and independently of it. The later stages of cognitive processing of targets were comparable between LSA and HSA but SAD modulated task performance by slowing the response latencies. This slowdown appears to be directly associated with the augmentation of evaluation fear symptoms. To conclude, these results argue for a similar processing of postures and faces, but also for the distinct influence of social anxiety in two stages of cognitive processing of social cues. These hypotheses have to be tested in further experiments to better understand the influence of the different facets of social anxiety on cognitive processing.
